# Supplementary material for: Selective atomic sieving across metal/oxide interface for super-oxidation resistance
Source: Nat Commun. 2024 Jul 21;15:6149. doi: 10.1038/s41467-024-50576-7 (PMC11271475; doi:10.1038/s41467-024-50576-7)
Supplement: Supplementary file 1 — Supplementary Information in Word format [file 41467_2024_50576_MOESM1_ESM.docx]

**Supplementary Information**

**Selective atomic sieving across metal/oxide interface for super-oxidation resistance**

Shuang Li^1#^, Li Yang^2#^, Jijo Christudasjustus^3^, Nicole R. Overman^4^, Brian D. Wirth^2^, Maria L. Sushko^3^, Pauline Simonnin^3^, Daniel K. Schreiber^4*^, Fei Gao^5*^, Chongmin Wang^1*^

^1^Environmental Molecular Sciences Laboratory, Pacific Northwest National Laboratory, 902 Battelle Blvd, Richland, WA 99352, USA

^2^University of Tennessee Knoxville, Knoxville, TN, 37996, USA

^3^Physical and Computational Sciences Directorate, Pacific Northwest National Laboratory, 902 Battelle Blvd, Richland WA 99352, USA

^4^Energy and Environment Directorate, Pacific Northwest National Laboratory, 902 Battelle Blvd, Richland, WA 99352, USA

^5^Department of Nuclear Engineering and Radiological Sciences, University of Michigan, Ann Arbor, MI, 48109, USA

# These authors contributed equally to this work

*Corresponding authors’ E-mail: [Daniel.Schreiber@pnnl.gov](mailto:Daniel.Schreiber@pnnl.gov), [gaofeium@umich.edu](mailto:gaofeium@umich.edu), [chongmin.wang@pnnl. gov](mailto:chongmin.wang@pnnl.gov)

**Table of Contents:**

1. Supplemental Electron Microscopy Data

2. DFT Calculations

3. Details of the PNP/cDFT Modeling

# Supplemental Electron Microscopy Data

## Analysis of Initial Ni-5Cr Sample

The starting condition of the tested Ni-5Cr sample is summarized structurally in Figure S1 and compositionally in Table S1. The initial TEM sample displays a ~2-3 nm thick native oxide layer, which was successfully removed by high-temperature vacuum annealing. Compositionally the sample was consistent with the nominal Ni-5Cr, with an average Cr concentration of 5.3 at.% as measured by STEM-EDS.


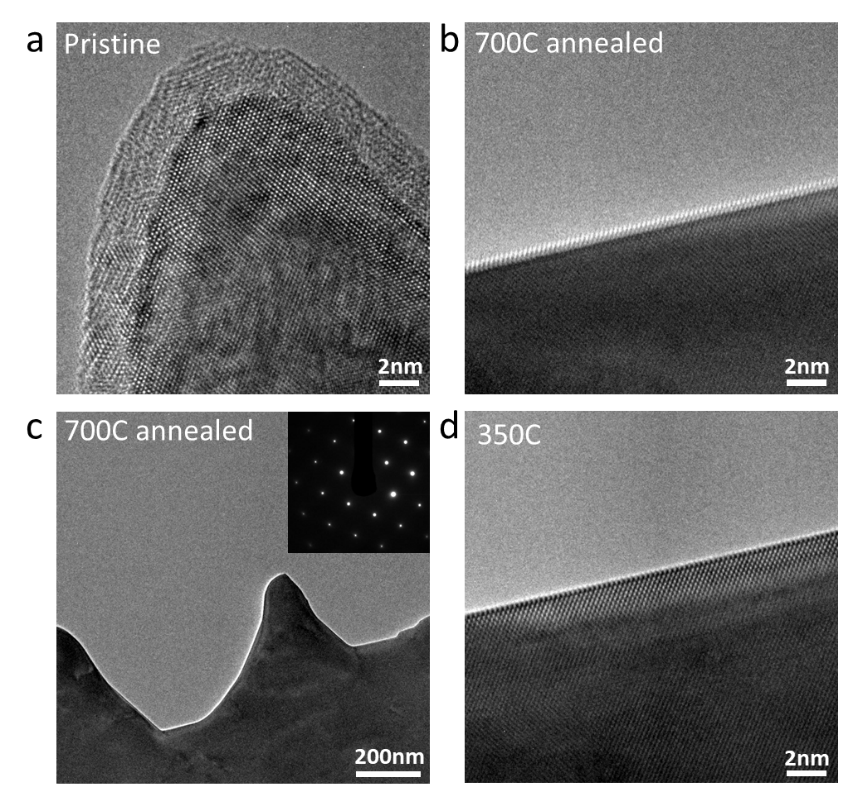


**Figure S1. Native oxide layer removed by high temperature annealing**. (**a**) HRTEM image of Ni-5Cr TEM specimen surface shows the native oxide layer with ~ 3 nm thickness. (**b, c**) The HRTEM image, bright-field image and electron diffraction patter acquired at elevated temperature (~700 ℃) after 30 mins annealing in high vacuum (~10^-7^ mbar), indicating the clean surface without native oxide layer after annealing. (**d**) The HRTEM acquired at 350 ℃, confirming the clean surface remains as the specimen temperature cool down in high vacuum.

**Table S1.** Energy dispersive X-ray spectroscopy elemental analysis of pristine Ni-Cr alloy shows an average of 5.3 at.% Cr.

| Element | Point1  (at%) | Point2  (at%) | Point3  (at%) | Point4  (at%) | Point5  (at%) | Point6  (at%) | Point7  (at%) | Point8  (at%) | Average  (at%) |
| --- | --- | --- | --- | --- | --- | --- | --- | --- | --- |
| Ni | 94.11 | 94.87 | 94.73 | 95.26 | 94.89 | 95.21 | 94.67 | 93.82 | 94.7 |
| Cr | 5.87 | 5.13 | 5.27 | 4.74 | 5.11 | 4.79 | 5.33 | 6.18 | 5.3 |

## In Situ Observation of Initial Oxidation Dynamics

Figures S2 and S3 display data corresponding to Figures 1 and 2 from the main manuscript without any supplemental annotations for a clearer presentation of the raw HRTEM data.

Figure S4 display the time-resolved HRTEM image of *in situ* oxidation at the (001) Ni-5Cr alloy surface in O_2_ with p = 1 × 10^−4^ mbar and T = 400 °C.

Figure S5 display the time-resolved HRTEM image of *in situ* oxidation at the (001) Ni metal surface in O_2_ with p = 1 × 10^−5^ mbar and T = 350 °C. We try to clarify the influence of Cr on oxidation behavior by carrying out *in situ* oxidation of Ni at (001) surface as edge and (110) as top/bottom surface. For pure Ni oxidation, as similarly observed for the case of Ni-5%Cr alloy, the upper/lower (110) surface of Ni metal is oxidized faster than (001) surface (images at 5.7s and 6s). The oxidation of (001) surface also follows the layer-by-layer oxidation with steps at the oxide/metal interface during initial state (at 9.4s). However, there is no two-layers morphology due to the absence of Cr. With the increased oxidation time, the oxidation evolved into 3D island growth. This (001) surface oxidation follows the Stranski-Krastanov (S-K) growth mode.


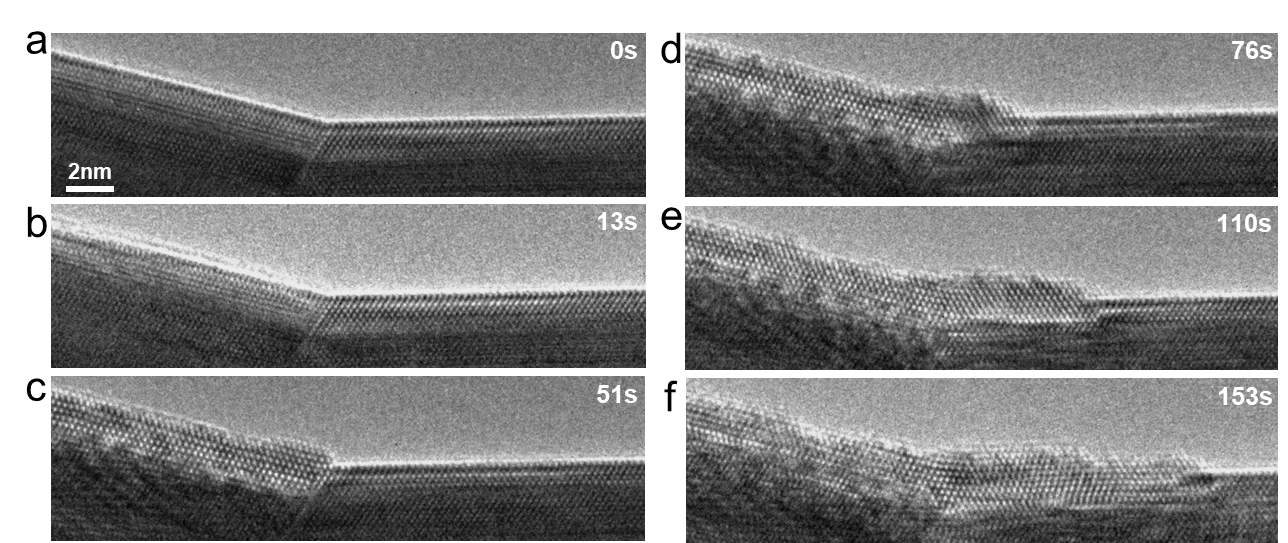


**Figure S2. The original time resolved HRTEM images of oxidation process on (111) and (001) alloy surfaces beside TB without any annotations.** (**a**) 0 s; (**b**) 13 s; (**c**) 51 s; (**d**) 76 s; (**e**) 110 s; (**f**) 153 s.


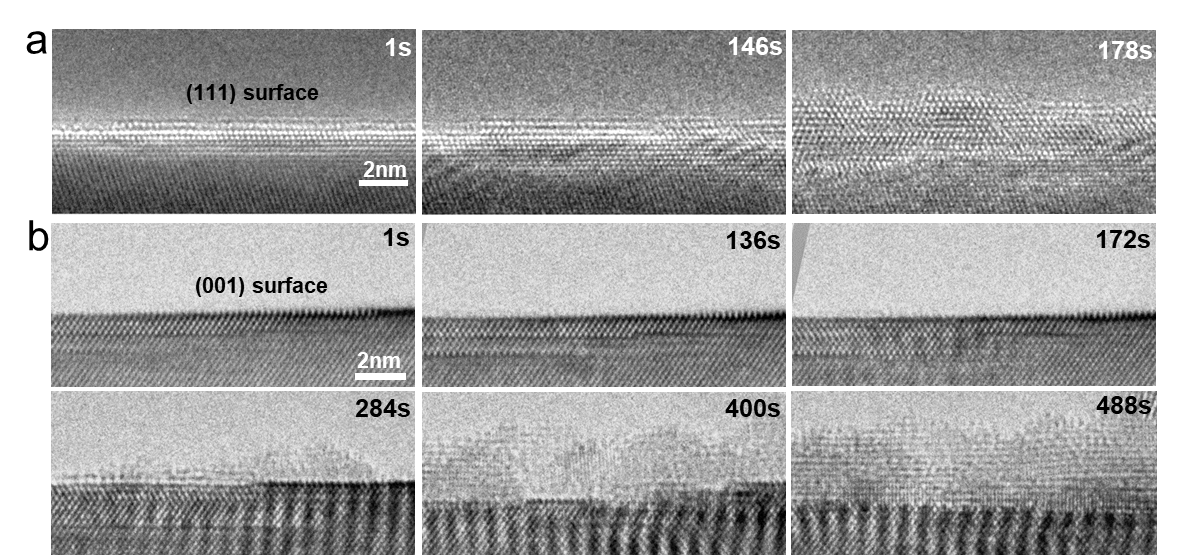


**Figure S3. The original time resolved HRTEM images of oxidation process on (111) and (001) alloy surface without any annotations.** (**a**) Three frames of images show the oxide evolution on (111) surface at time of 1s, 146 s, and 178 s; (**b**) Six frames of images show the oxide evolution on (001) surface at time of 1s, 136 s, 172 s, 284 s, 400 s, and 488 s;


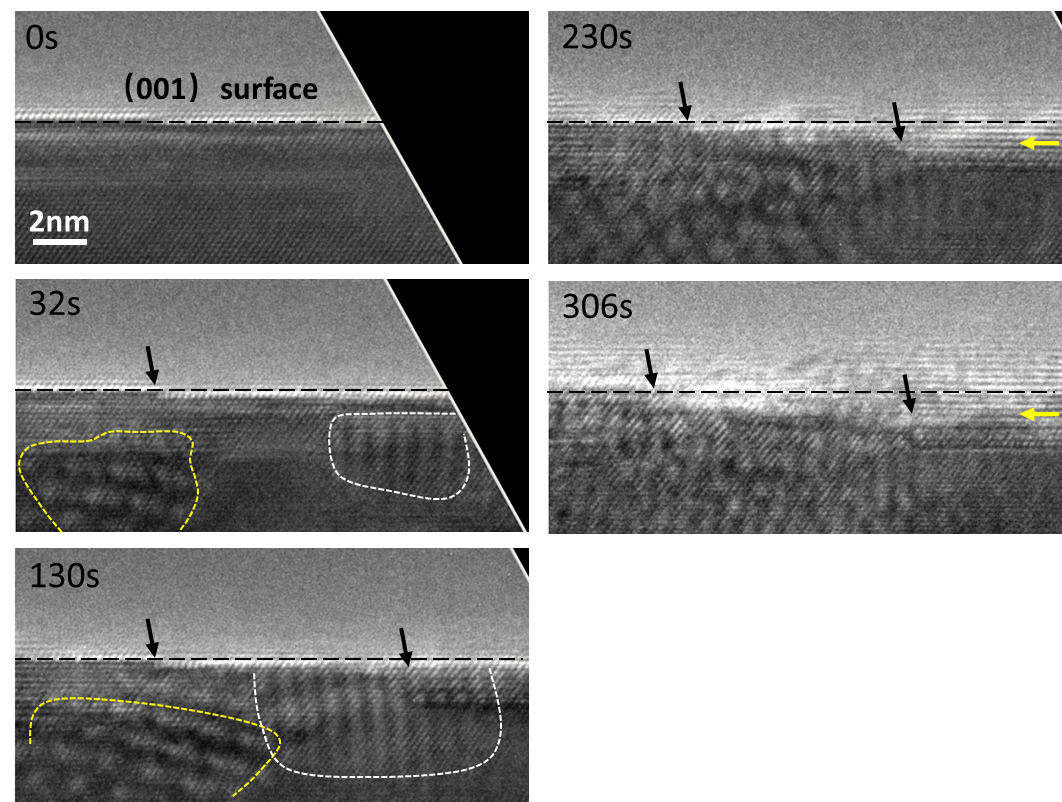


**Figure S4****.** **Time-resolved HRTEM images reveal the growth of the oxide layers at the (001) Ni-5Cr alloy surface in O_2_ with p = 1 × 10^−4^ mbar and T = 400 °C.** The black dash lines indicate the original alloy surface position. The white and yellow dash line marks the oxide on the upper or lower (110) surface. The black arrows indicate the steps at the alloy surface. The yellow arrows indicate the possible interface between the inner and outer oxide layer.


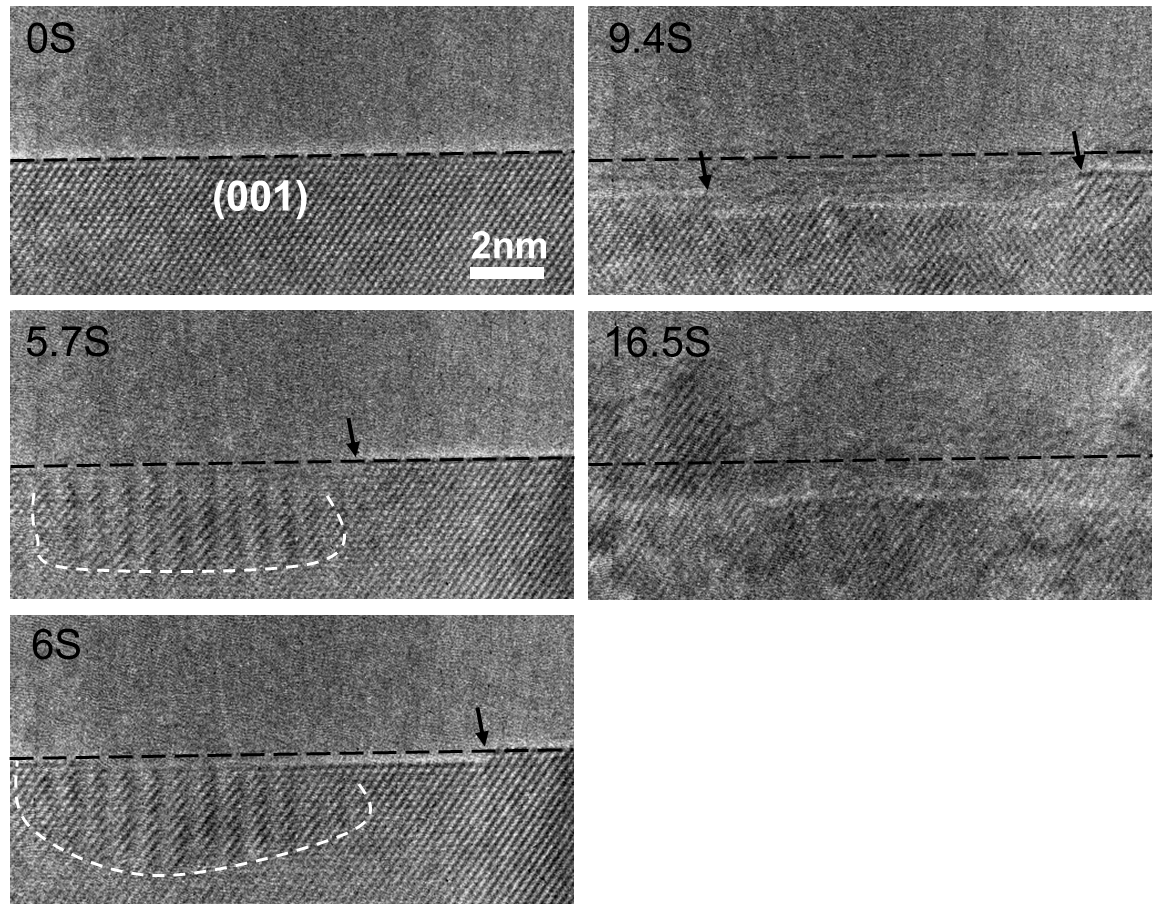


**Figure S5. Time-resolved HRTEM images reveal the growth of the oxide layers at the (001) Ni metal surface in O_2_ with p = 1 × 10^−5^ mbar and T = 350 °C.** The black dash lines indicate the original alloy surface position. The white dash line marks the oxide on the upper or lower (110) surface. The black arrows indicate the steps at the alloy surface.

## Oxidation-Induced Structural and Chemical Evolution of Alloy Surfaces

Figure S6 presents STEM EELS elemental intensity profiles corresponding to Figure 2c, e in the main document. Figure S7 and Figure S8 present further HRTEM, FFT images, corresponding orientation relationships (ORs) and Moiré fringes analysis for the oxide/metal interfaces.

The Moiré fringes generated on either the (001) side surface sample and (111) side surface sample are believed to be induced by the of the slight difference in the interplanar spacing of Ni and NiO generated on the upper/lower (110) surface. For the Moiré fringes induced by different interplanar spacing without rotation, the Moiré fringes spacing can be calculated by the equation: $D=\frac{d_{1}d_{2}}{d_{1}-d_{2}}$ .

For the Moiré fringes in Figure S7, the (220) interplanar spacing of NiO and Ni were measured from the FFT as 0.124 nm (d_1_) and 0.146 nm (d_2_), while the Moiré fringes spacing was measured as 0.83 nm (D). They are consisting with the equation.

For the Moiré fringes in Figure S8, the (11-1) interplanar spacing of NiO and Ni were measured from the FFT as 0.241 nm (d_1_) and 0.204 nm (d_2_), while the Moiré fringes spacing was measured as 1.35 nm (D). They are consisting with the equation.


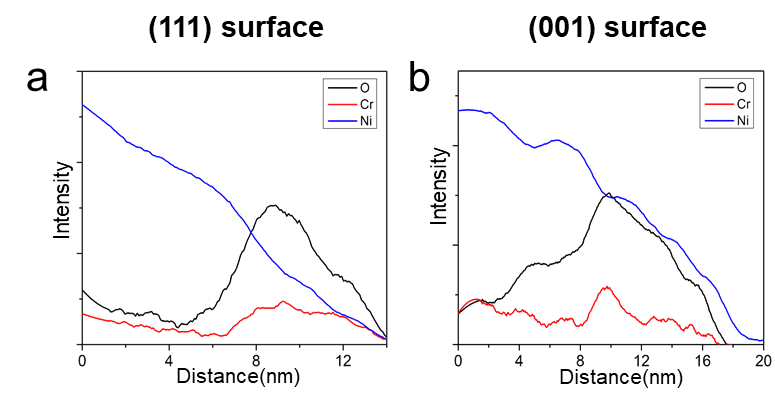


**Figure S6. The element intensity line profile from the corresponding EELS line scan in Figure 2c, e.** (**a**) On the (111) surface; (**b**) on the (001) surface. It should be noted that the element intensity line profile of (001) surface (b) shows the oxide layer surface sit at distance ~17.5 nm with nearly zero intensity, which implies the element concentration beyond 17.5nm is due to the wrong calculation with the noise signal, therefore we only display the element concentration till 17.5nm in Figure 2e.


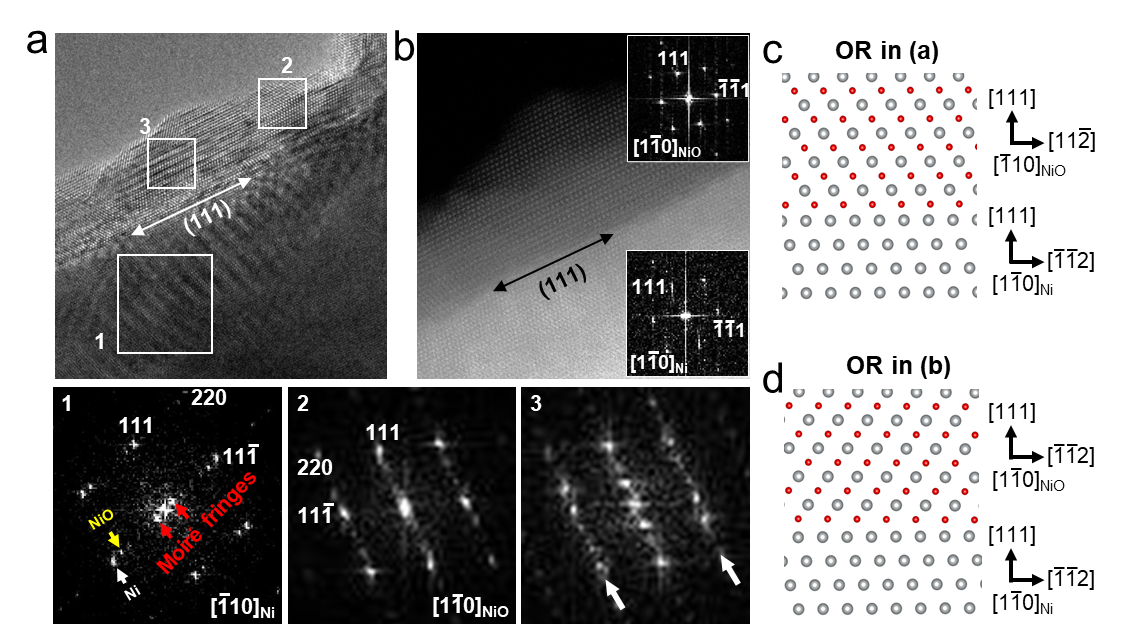


**Figure S7. Lattice structure analysis of the Cr-rich NiO layers at (111) surface of Ni-Cr alloy.** The images are acquired from the area not exposed to the electron beam during oxidation. The FFTs of the region marked as “1, 2” in HRTEM image (**a**) are consistent with the Ni/NiO with [-110] zone axis and NiO with [1-10] zone axis. The line feature in FFT of the region “3” indicate the high density of stacking faults. The insert FFTs in (**b**) indicate the Ni and NiO with [1-10] zone axis. The (**c**) and (**d**) show the OR between NiO and Ni in (a) and (b), respectively. They both consist with the cubic-on-cubic OR (NiO{111} //NiCr{111}, NiO<110> //NiCr<110>) .


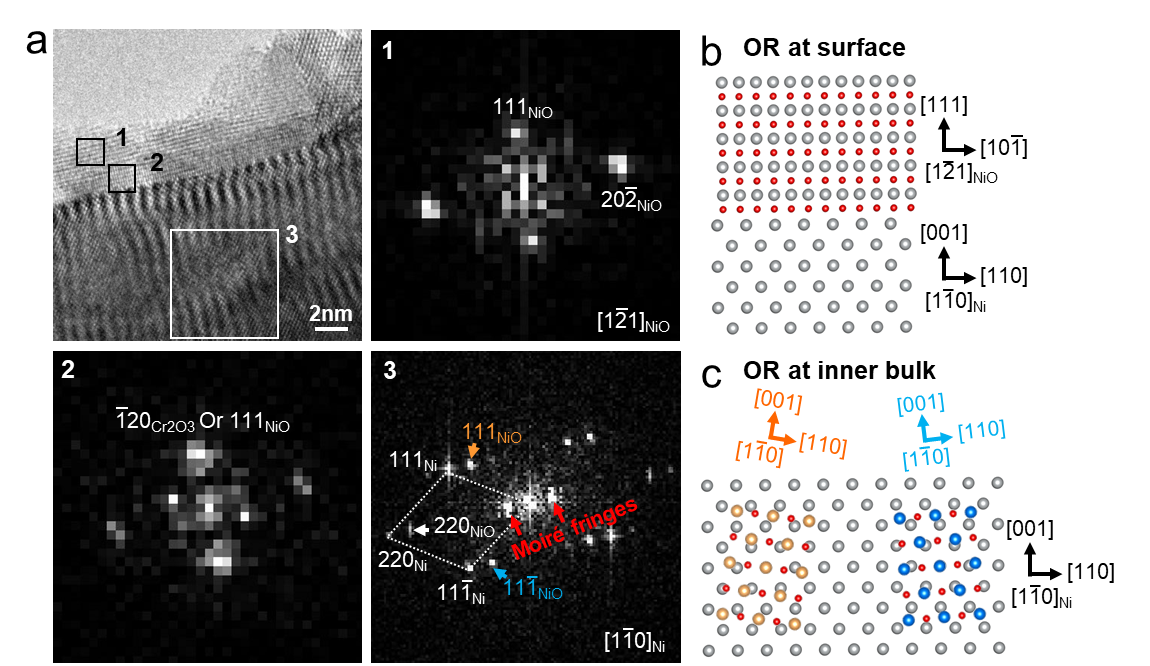


**Figure S8.** **Lattice structure analysis of the two oxide layers at (001) surface of Ni-Cr alloy.** (**a**) The FFTs of the region marked as “1, 2” in HRTEM image are consistent with the NiO with [1-21] zone axis and Cr_2_O_3_ with [010] out-of-plane orientation. The FFT of region “3” indicate the NiO oxides grown on the upper and lower surface of the inner bulk alloy. The FFTs of region “1” and “3” indicate the NiO grown on the upper and lower surface of the inner bulk alloy have the different orientation relationships with the alloy compared to the oxides grown on the (001) edge surface. The (**b**) and (**c**) show the OR between NiO and Ni on (001) edge surface (“1” FFT) and bulk alloy (“3” FFT), respectively.

## Evaluation of Electron Beam Effect

The safe electron dose rates for environmental TEM studies have been established in the previous published literatures outlined in this section. For the case of metal nanoparticulate catalysts supported on an oxide (Au/CeO_2_), an electron dose rate of ~2.5×10^5^ e/nm^2^·s and a pressure of ~ 0.45 mbar were used to visualize the gas molecules adsorbed on Au surface^1^. In this work, an electron dose rate of ~5×10^4^ e/nm^2^·s and a gas pressure of ~1×10^-4^ mbar is used even though the alloy sample used here is less sensitive to electron beam artifacts than nanoparticulate catalysts. Similar experimental parameters (electron dose rate ~5×10^4^ e/nm^2^·s, pressure ~ 1×10^-5^ mbar) had been used for the case of Ni-Cr thin film (~50 nm in thickness) in previous work of our group, which similarly confirmed no visible electron beam modification during the *in situ* HRTEM imaging^2^. In addition, we have evaluated the beam effect on our oxidation experiments by observing the area that is not exposed to the electron beam during *in situ* oxidation. Images in Figure S7 and Figure S9 are taken from areas that are not exposed to the electron beam, where the oxide layers on (111) and (001) alloy surface are consistent with the *in situ* observations in Figure 2, respectively. This indicates that the crystallographic-dependent oxidation behavior does not derive from an electron beam artifact.


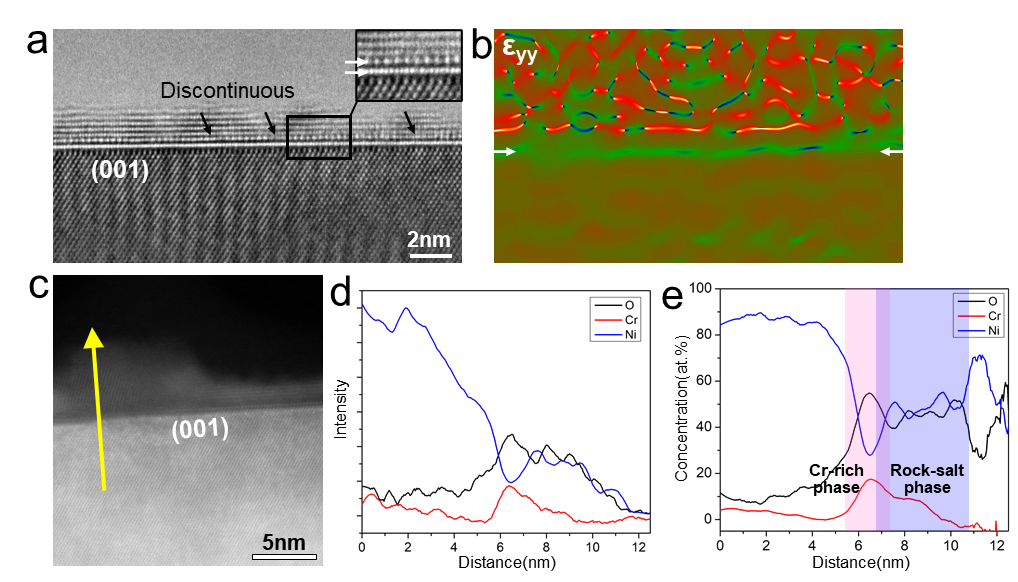


**Figure S9. Two oxide layers at the (001) surface after initial oxidation, which is acquired from the area not exposed to the electron beam during oxidation.** (**a**) The HRTEM image of the two oxide layers at the (001) surface. The white arrows in insert enlarged image clearly indicate the one atomic inner oxide layer with different structure. The black arrows indicate the inner oxide layer is discontinuous. (**b**) The out-of-plane strain distribution gained by geometric phase analysis of (a). The white arrows indicate the sharp changes near the oxide-alloy interface. (**c-e**) The HAADF-STEM images of the outer (rock-salt phase, i.e., NiO) and inner (Cr-rich phase) oxide layers and the relative element intensity and concentration line profile obtained from EELS line scan along the yellow line.

## Structural and Chemical Information of Oxide Phases after Long-Term Oxidation

Figure S10 presents STEM EELS intensity line profiles corresponding to Figure 3(b) and (f) of the main manuscript. Figure S11 shows HRTEM images of chromia and NiCr_2_O_4_ phases formed during longer-term *in situ* oxidation of the Ni-5Cr sample. Figure S12 shows the oxide formed on the surface of the twin boundary region of the sample reported in Figure 1 of the main manuscript after long term oxidation (12 and 37 min), which depicts a relatively comparable oxide thickness at these extended times.


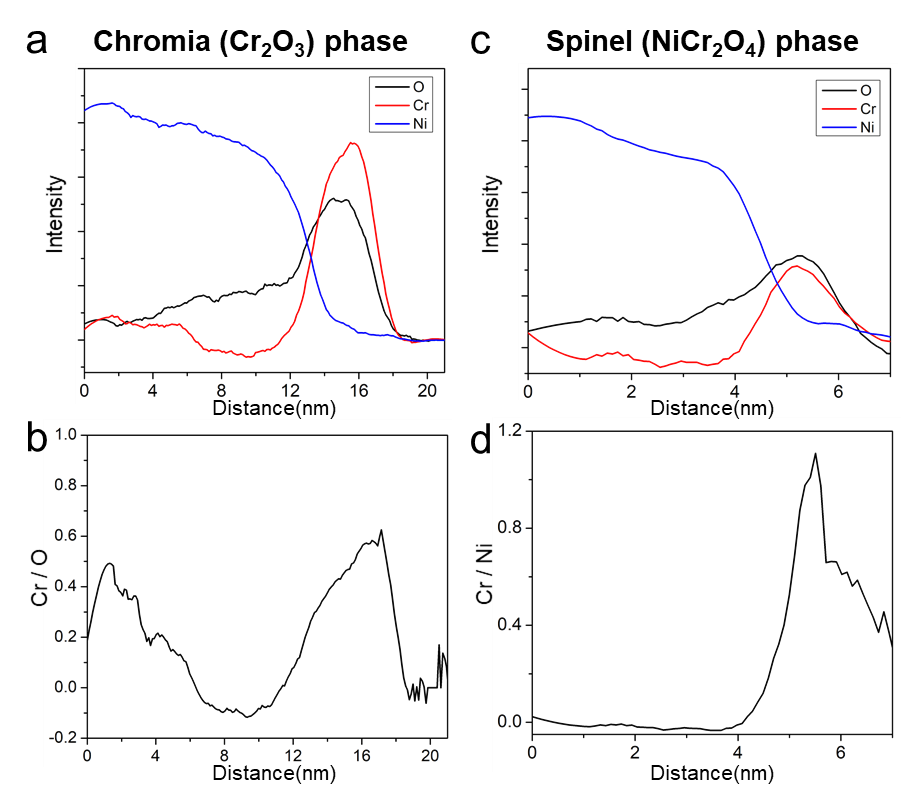


**Figure S10.** **The element intensity and Cr/O (or Cr/Ni) ratio line profile from the corresponding EELS line scan in Figure 3(b) and (f).** (**a**) Chromia (Cr_2_O_3_) phase; (**b**) The Cr/O ratio increase to about 0.6 in the oxide layer, close to the 0.67 in chromia phase. The Cr/O ratio also seems high in the bulk region may be due to the noise of the low intensity of Cr and O in the bulk region; (**c**) Spinel (NiCr_2_O_4_) phase; (**d**) The Cr/Ni ratio below surface is less than 0.05, which implies the below surface is Cr-lack compared to the original alloy composition. It increases to about 1.1 in the oxide layer. The ratio does not reach 2 as in the stoichiometric ratio may associate with the disorder structure or the overlap of spinel and rock-salt oxides.


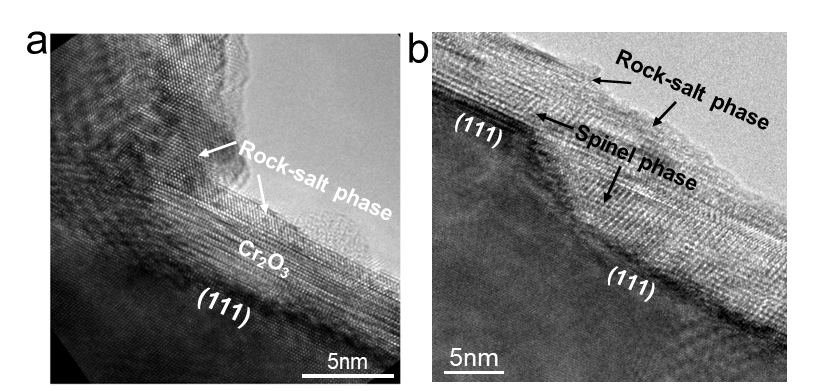


**Figure S11.** **The HRTEM images of chromia and spinel phase generated at the (111) surface during extended oxidation.** (**a**) a region of (111) surface; (**b**) another region of (111) surface.


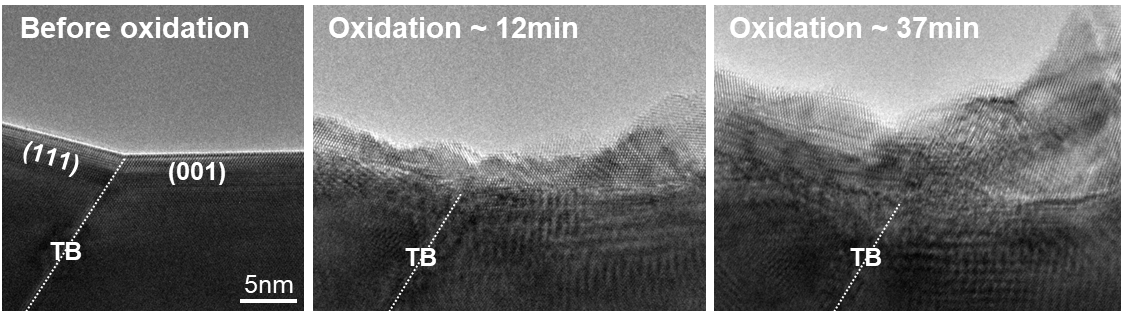


**Figure S12.** **In situ ETEM observation of oxidation of the twin boundary region described in Figure 1 of the main manuscript after 12 min and 37 min.**

# DFT Calculations

Considering the accuracy and feasibility, the energy cutoff for plane waves in the surface and interface calculations is 500 and 400 eV, respectively. The convergence criterion for the electronic self-consistent iteration is 10^-5^ eV for all calculations, but the force convergence criteria for the unfixed atoms is 0.02 and 0.05 eV/Å respectively for the surface and interface. Brillouin zone integrations were performed using Monkhorst-Pack grids with 6×6×1 or 4×4×1 for surface and interface, respectively. The surface or interfacial planes were constrained to the calculated Ni lattice constant.

## Calculation of Ni surface

The lattice constant of fcc Ni is 0.35177 nm. A nine or ten-layer slab with three bottom layers frozen and a ~ 1.5 nm vacuum layer was used for the Ni(111) and Ni(100) surface, respectively. Each atomic layer for both surface slabs contain 4 Ni atoms.

### **Oxygen adsorption energy on the Ni surface**

The most stable adsorption site on the Ni(111) and (001) surfaces for an oxygen atom is referred to as ‘fcc’ and ‘4f’ sites (Figure S12), respectively, which is consistent with the previous work^3^. The average adsorption energy per O atom is defined as

$E_{O}^{ads}=(E_{Ni}^{tot}+nE_{O_{2}}/2-E_{Ni+nO}^{tot})/n$ (1)

where $E_{Ni+nO}^{tot}$ and $E_{Ni}^{tot}$ are the total energies of the surface system with and without O atom. *n* is the number of adsorbed O atoms.$E_{O_{2}}$is the energy of a di-oxygen molecule. A positive adsorption energy means that O atoms are energetically adsorbed on the surface.

### **Ni diffusion energy near the Ni surface**

Ni diffusion activation energy near the Ni(111) surface covered with 1ML O has been calculated by CI-NEB method. Three images were inserted between the initial and finial states. The Ni migration near the Ni(001) covered with 1 ML O is not considered since the finial state, i.e. the Ni atom that comes from the first Ni layer, cannot stably reside on the top of the oxygen layer.

### **O diffusion energy near Ni surface**

For the O coverage of 0.25 ML, the activation energy for O diffusion from the most surface adsorption site towards bulk Ni has been investigated. The relatively stable sites below Ni surface are octahedral interstitial sites (OISs). Three or five images are inserted between two nearby stable states.

### **Discussion**

The positive adsorption energies for oxygen on both Ni(111) and (001)surfaces mean that the adsorption process is energetically favorable (Figure S13a). The Ni(111) with higher energy implies that oxygen adsorption is easier on Ni(111) surface, which is consistent with the *in situ* observations of incipient oxidation. As the coverage of oxygen increases, the diminishing adsorption energies implies that further adsorption become sluggish.

In addition to oxygen adsorption, the first oxide layer nucleation requires either outward diffusion of Ni or inward diffusion of O into Ni metal. For the surface with fully covered oxygen, the outward Ni diffusion near the (111) surface can occur with an energy of 0.81 eV (Figure S13b). This process is unlikely to be observed for the (001) surface since Ni could not stably reside on the top of the adsorptive oxygen layer. It should be one reason for the slower oxide nucleation of the (001) surface. Notice, it does not mean the Ni diffused out will not happen at all on (001) surface because we have not considered the diffusion at the step edges on the not fully covered surface.

The total energy barriers of O atom diffusion from the surface into the second layer of bulk metal are relatively high on both the (111) and (001) surface (Figure S13c), as 4.04 and 3.63 eV, respectively. The average kinetic energy of molecules at above 1000 K will be sufficient to overcome these barriers, which is higher than our experimental temperature. Therefore, the oxide nucleation reactions with oxygen diffusion at our experiment condition are considered as low temperature oxidation controlled by kinetics. Instead of the total energy barrier, the energy barrier of the first jumping event is most important in determining the reaction rate. The oxygen adsorbed on the (111) surface can more easily penetrate into the subsurface than on the (001) surface because of the lower energy barrier of the first jump (2.43eV for (111), 3.17 eV for (001)). In addition, the lower diffusion barrier of the first jump also could be deducted by the straight diffusion path from the ‘fcc’ adsorption site on (111) surface, instead of the step path on (001) surface, which goes across tetrahedral interstitial to octahedral interstitial positions, as indicated by black arrows in Figure S13d. This higher oxygen diffusion barrier of first jump should be the other reason for the slower oxide nucleation of (001) surface.


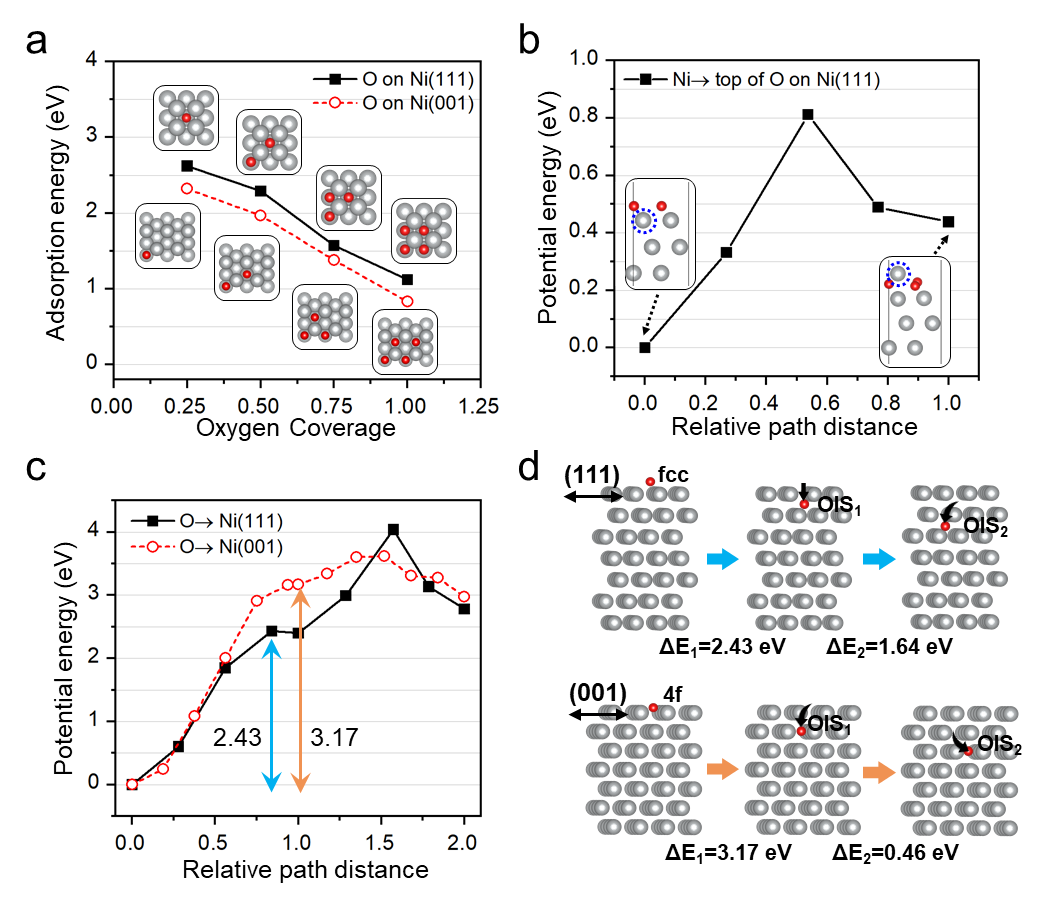


**Figure S13.** **DFT calculation of the oxygen adsorption energy and diffusion energy barriers for O and Ni atoms on the (111) and (001) surface.** (**a**) Adsorption energy of O atom as a function of oxygen coverage on the surface. (**b**) Activation energy for diffusion profile of one Ni atom diffused from bulk to the top of adsorption oxygen layer on (111) surface. (**c**) Activation energy for diffusion profile of one adsorptive O atom diffused from surface into Ni metals. (**d**) The atomic models show adsorptive O atom diffusion paths (indicated by black arrows) and corresponding energy barriers in (c).

## Calculation of O and Ni diffusion across the NiO/Ni interface

The lattice constants for fcc Ni and rocksalt NiO are 0.3479 and 0.4191 nm predicted using GGA+U with U_eff_ = 4.3 eV. AF2 antiferromagnetic spin structure is applied for NiO. Supercells for NiO/Ni interfaces contain 4 Ni ions per metal layer and include ~1.5 nm vacuum space. The NiO(111)/Ni(111) and NiO(111)/Ni(001) interface contains 8 and 9 Ni layers in the fcc Ni site, and both have three metal layers and four oxygen layers in the NiO side. In consideration of the stability of NiO(111) surface^4^ and oxygen-rich condition, an oxygen terminated octopolar structure is created on the surface side for NiO.

**Table S2.** **Energy barriers (in eV) of the O atom and Ni vacancy cross the interface from Figure 4.** The energy barrier of I_O_ diffuse forward cross NiO(111)/Ni(111) interface is zero when the O half atomic layer away from the interface, increased to 0.4 when the O atom one atomic layer away from the interface. The ‘–’ represent the zero or near zero energy barrier, which means it will react spontaneously.

|  | I_O_ (from NiO to Ni) | | V_Ni_ (from NiO to Ni) | |
| --- | --- | --- | --- | --- |
|  | forward | back | forward | back |
| NiO(111)/Ni(111) | ̶ or 0.4 | 4.69 | ̶ | 1.24 |
| NiO(111)/Ni(001) | 0.54 | 4.86 | 0.82 | 1.01 |

## Mathematical analysis according to the simulated energy

The jump frequency of the diffusion step is defined as:

$\Gamma=\Gamma_{0}exp\left( -\frac{E^{diff}}{kT} \right)$ (2)

The rate of the element to go through the interface is the rate of passing the forward energy barrier without going back, which corresponds to the following rate:

$\Gamma_{inter}=\Gamma_{for}(1-\Gamma_{back})$ (3)

Therefore, the jump frequency of the I_O_ (or V_Ni_) can be written as:

$\Gamma_{inter}=\Gamma_{0}exp\left( -\frac{E_{1}}{kT} \right)-\Gamma_{0}exp\left( -\frac{E_{2}}{kT} \right)$ (4)

Where the E_1_ is the energy barrier for diffusion forward and E_2_ is the energy barrier for diffusion backward. For the NiO(111)/Ni(001) interface, the E_1_ and E_2_ for I_O_ are 0.54 and 4.86 eV, for V_Ni_ are 0.82 and 1.01 eV. Therefore, the Γ_IO_ is larger than Γ_VNi_, which means the jump frequency of oxygen diffuses across the NiO(111)/Ni(001) interface is higher than Ni vacancy.


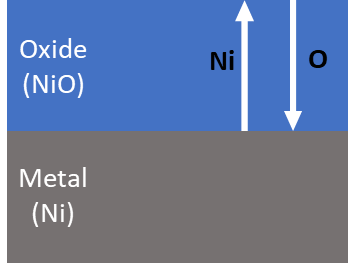


**Figure S14.** **Schematic shows the O and Ni diffusion paths during oxidation.**

The whole oxidation path requires O to diffuse into the metal from the surface and Ni to diffuse to surface from the metal (both across the oxide layers, as schematic in Figure S14). Therefore, the diffuse jump frequency for the whole path including the diffusion across the interface and within the oxide as:

For (111) surface:

$\Gamma_{Ni}^{111}=\Gamma_{Ni in NiO}\Gamma_{Ni inter}^{111}=\Gamma_{Ni in NiO}$ (5)

$\Gamma_{O}^{111}=\Gamma_{O in NiO}\Gamma_{Ni}^{111}+\Gamma_{O in NiO}\Gamma_{O inter}^{111}$

$=\Gamma_{O in NiO}\Gamma_{Ni in NiO}+\Gamma_{O in NiO}\Gamma_{O inter}^{111}$ (6)

Where the $\Gamma_{Ni inter}^{111}$ is 1 due to no energy barrier; the $\Gamma_{O}^{111}$ containing two terms due to the interstitial O and CVC migration mechanisms, respectively.

For (001) surface:

$\Gamma_{Ni}^{001}=\Gamma_{Ni in NiO}\Gamma_{Ni inter}^{001}$ (7)

$\Gamma_{O}^{001}=\Gamma_{O in NiO}\Gamma_{O inter}^{001}$ (8)

To compare the O/Ni diffusion ratio on each surface by calculate the jump frequency ratio of O vs Ni, as:

$\frac{{\Gamma_{O}^{111}}/{\Gamma_{Ni}^{111}}}{{\Gamma_{O}^{001}}/{\Gamma_{Ni}^{001}}}=(\Gamma_{Ni in NiO}+\Gamma_{O inter}^{111})\frac{\Gamma_{O inter}^{001}}{\Gamma_{Ni inter}^{001}}$ (9)

Where the $\Gamma_{O inter}^{111}$ is approximated to be 1 because there is no energy barrier for I_O_ diffuse forward when the O half atomic layer away from the interface, as in Table S2. The $\frac{\Gamma_{O inter}^{001}}{\Gamma_{Ni inter}^{001}}$ is large than 1 as the discussion above.

Therefore, we deduce that the O/Ni diffusion ratio on (111) surface is higher than on the (001) surface, which well explains the hierarchical oxides on (001) surface due to the slow O diffusion rate.

## Role of Cr at the interface.

The lattice constant for rocksalt NiO is 0.4191 nm as predicted using GGA+U with U_eff_ (Ni)= 4.3 eV and U_eff_ (Cr)= 4.0 eV. AF2 antiferromagnetic spin structure is applied for NiO. In the presence of Cr, we extended the size of the supercell to a 4*4 supercell and include ~1.5 nm vacuum space. The NiO(111) have five metal layers and 6 oxygen layers. The octopolar structure has been disregarded in this case, as the bottom 3 layers of atoms are frozen to represent the bulk rocksalt. The other interface has the oxygen coverage of the NiO/Ni interface.

To understand the relevant transport kinetics, the effect of Cr at the interface can hardly be ignored. However, it would require an extensive amount of calculations to truly grasp Cr involvement in the diffusion and migration mechanisms between Ni and rocksalt interfaces. On one hand, recent work at the Ni(100) free surface in dilute NiCr alloy showed that the mechanism of diffusion of a vacancy from bulk to the surface should result in a backward flux of Cr in the atomic plane just below the surface^5^. In turn, the presence of Cr below that free surface affect in-plane migration jump preventing vacancies to sit on top of surbsurface Cr^5^.

On the other hand, our preliminary calculations have shown that the migration barrier of Cr into a Ni vacancy of reverse spin is 4.3 eV. This is consistent with previous observations of the diffusion barrier of a charge neutral Ni vacancy in reverse spin in bulk NiO of about 2.53 eV^6^, value that is clearly influenced by the charge of the vacancy and the spin orientation of the rocksalt^7, 8^. Cr residing in the NiO lattice in the position of a cation vacancy is favorable, as shown in Figure S15, although the energy difference is almost negligeable.

It appears that Cr plays a crucial role into the vacancy diffusion mechanism as it does not allow vacancies to be trapped at the Ni free surface, whereas in the rocksalt, vacancy migration toward the free surface is favorable. Competing mechanisms, as well as exhaustive diffusion paths, should be further investigated to better understand the role Cr has to play in the sieving mechanism at the interface.


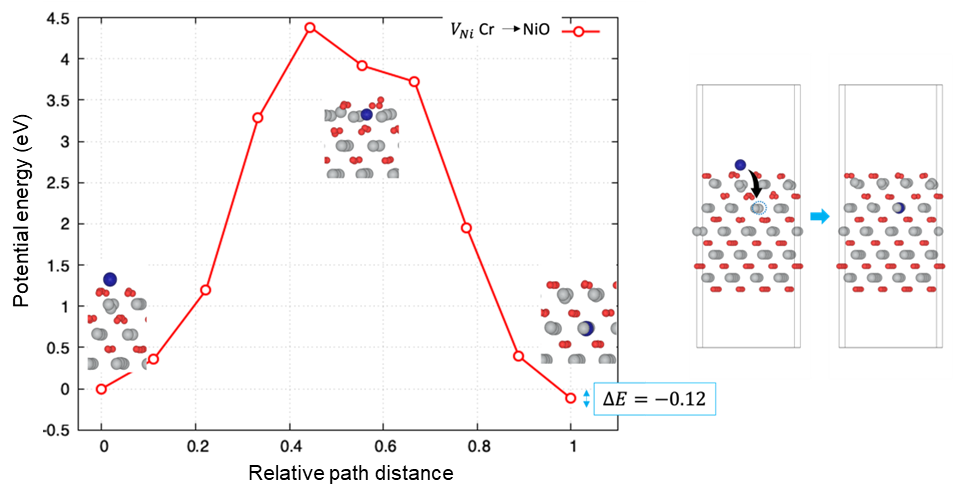


**Figure S15.** **DFT calculation of the chrome diffusion energy barriers NiO(111) surface.** Diffusion energy profile of one Cr diffusing into a Ni vacancy in the bulk of NiO. The atomic models show the distortion of the solid for the migration of Cr.

# Details of the PNP/cDFT Modeling

Classical density functional theory (cDFT), within the context of the Poisson-Nernst-Planck (PNP) transport kinetics model, was used to further simulate the oxidation process of the alloy. The model employs a steady state formulation of the PNP. In this formulation, the fluxes *J_i_* for matrix and minor elements in the metal alloy and for oxidizing species satisfy the Nernst equation (10) coupled to the Poisson equation (12) and the steady state condition (11):

$-J_{i}=D_{i}\left( r \right)\left[ \nabla\rho_{i}+\frac{1}{k_{B}T}\rho_{i}\left( q_{i}e\nabla\varphi+\nabla\mu_{i}^{id}\left( r \right)+\nabla\mu_{i}^{ex}\left( r \right) \right) \right]$ (10)

$\nabla J_{i}=0$ (11)

$-\nabla\left( \varepsilon\left( r \right)\nabla\varphi\right)=4\pi\left( \rho_{f}\left( r \right)+e\sum_{i} q_{i}\rho_{i} \right)$ (12)

In these equations *D_i_(r)* and *r_i_* are the diffusion coefficients and densities, respectively, of all the species, $\varphi$ is the electrostatic potential, *m*^id^ and *m*^ex^ are the ideal and excess chemical potential, respectively, *r_j_(r)* is the fixed charge density in the system (if any), *k_B_T* is the thermal energy, and *e* is the electron charge. Index “*i*” denotes mobile species type, e.g. for CrNi/oxide interface *i=1* corresponds to Ni, *i=2* to Cr, and *i=3* to O. cDFT is used for evaluation of the chemical potentials of all species and the total free energy is divided into two parts: the ideal part (*F*^id^), which includes the contributions from the configurational entropy of the non-interacting species and bonding enthalpy (if any), and the excess free energy, which has contributions from all interactions in the system. These include the free energies of Coulomb interactions, electrostatic correlations, hard sphere repulsion, and short-range interactions with the stationary (lattice) sites, which represent the equilibrium sites for matrix and minor elements in the crystal structure of the alloy and oxide^9, 10^. The model allows any number of mobile species, which makes it suitable for modeling not only coupled metal atom and oxygen transport and reactions in static crystal lattices, but also to include an explicit treatment of vacancy migration. Metal and oxygen ions are modeled as hard spheres with the diameters equal to the corresponding Pauling ionic diameters. To reach into experimentally relevant length scales of hundreds of nanometers, the position of each individual ion in 3D space is not recorded. Instead, 3D distributions of all species are represented as density maps on a grid with the spacing equal to a fraction of interatomic distances (1/5 in this work). This way we efficiently capture collective phenomena at scales that can be measured experimentally, without tracking the dynamics of each individual ion.

A metal alloy/oxide interface is modeled in 3D as an array of interaction centers representing atomic positions of Ni, Cr, and O in the crystal lattices of the alloy (fcc), NiO and Cr_2_O_3_ oxide phases. The crystallographic vectors of the Ni fcc lattice are aligned along the Cartesian vectors of the simulation cell. An oxide slab of finite thickness (3 nm) resides at the planar surface of the metal alloy, and the oxide slab is truncated at one end to represent the alloy/oxide interface at the leading edge of the oxidation front. The lattice of interaction centers in the oxide slab corresponds to a superposition of Ni atomic positions in NiO and Cr atoms in Cr_2_O_3_ oxides. The assumption was made that the oxygen concentration in the oxide region is sufficient for forming stoichiometric metal oxides with NiO and Cr_2_O_3_ structures. The oxide phases were selected to match previous experimental observations of stable oxide microstructures^11, 12^. The structure of a Ni-5Cr alloy was constructed by substituting 5 at.% Ni by Cr in the metal matrix.

The total excess free energy functional is expressed as:

$F^{ex}=F_{C}^{ex}+F_{el}^{ex}+F_{hs}^{ex}+F_{sh}^{ex}$ (13)

All contributions to the excess free energy, except the short-range term, are calculated from first principles using the Fundamental Measure Theory and Mean Spherical Approximation. The Coulombic term is calculated through the solution of the Poisson equation (3) and is included in (1) as $q_{i}e\nabla\varphi$ contribution. The short-range interactions between mobile species (denoted as “m”) and the equilibrium sites (denoted as “s”) in the crystal lattice of alloy and oxide are given by

$F_{sh}^{ex}=\frac{1}{2}\int_{\Omega} \int_{\Omega} drdr^{'}\sum_{\alpha,\beta=m,s} \rho_{\alpha}\left( r \right)\rho_{\beta}\left( r^{'} \right)\Phi_{\alpha\beta}\left( \left| r-r^{'} \right| \right)$(14)

where $\Phi_{\alpha\beta}\left( \left| r-r^{'} \right| \right)$ is the square-well potential

$\Phi_{\alpha\beta}\left( \left| r-r^{'} \right| \right)=\left\{ \begin{aligned} -\tau, \left| r-r' \right|<{1.2\sigma}_{\alpha\beta} \\ 0, \left| r-r' \right|\geq{1.2\sigma}_{\alpha\beta} \end{aligned} \right.$ (15)

with $\sigma_{\alpha\beta}$ equal to the contact distance between species *a* and *b* and depth *t* equal to the barrier for the elementary diffusion processes in alloy region ($E_{\alpha}$), which were calculated using plane-wave DFT^13^, and equal to formation energies of various possible oxide phases in the oxide region ($H_{\alpha}$). In this way, our simulation approach retains a high accuracy treatment of the dynamics of elementary transport processes and uses this information to set rate parameters in the PNP/cDFT simulation.

Short-range interactions in oxide region are characterized by the formation energies, equal to *H_Cr_* = *H_O_* = -11.7 eV for Cr_2_O_3_ and *H_Ni_* = *H_O_* = -2.5 eV for NiO^14-16^. Experimental values for the diffusion coefficients of mobile species were used with *D_Cr_* = 5.21×10^-13^ cm^2^/s, and *D_Ni_* = 2.92×10^-13^ cm^2^/s^17^. Simulations were performed at 350 °C matching the temperature employed in the experimental work.


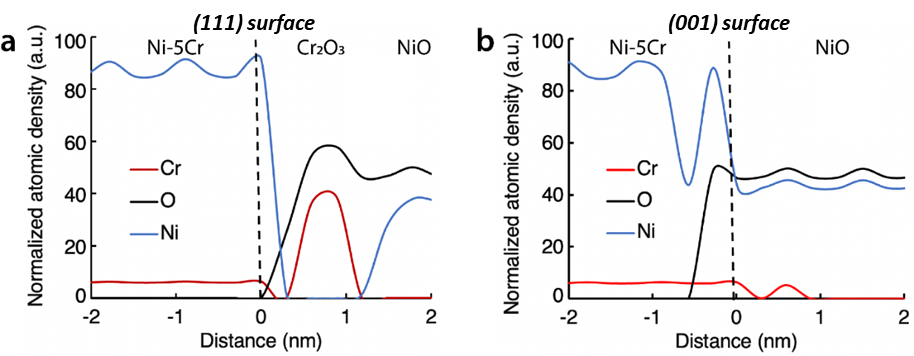


**Figure S16.** **Simulated** **density distribution at Ni-5Cr interface during oxidation of (111) and (001) surface.** (**a**) On (111) surface; (**b**) on (001) surface. Zero distance corresponds to the initial position Ni-5Cr surface, Ni density is shown as bule line, O – black line, and Cr – red line.

**References**

1. H. Yoshida *et al.*, Visualizing Gas Molecules Interacting with Supported Nanoparticulate Catalysts at Reaction Conditions. *Science* **335**, 317-319 (2012).

2. L. Luo *et al.*, Atomic origins of water-vapour-promoted alloy oxidation. *Nat Mater* **17**, 514-518 (2018).

3. O. Olatunji-Ojo, C. D. Taylor, Changes in valence, coordination and reactivity that occur upon oxidation of fresh metal surfaces. *Philosophical Magazine* **93**, 4286-4310 (2013).

4. W.-B. Zhang, B.-Y. Tang, Stability of the polar NiO(111) surface. *The Journal of Chemical Physics* **128**, 124703 (2008).

5. J. Startt, C. Deo, R. Dingreville, Vacancy Surface Migration Mechanisms in Dilute Nickel-Chromium Alloys. *Scr. Mater.* **202**, 113998 (2021).

6. H. Sun, First principles calculation of charged vacancy diffusion in nickel oxide. *http://d-scholarship.pitt.edu/27315/ (accessed 2023-07-14).* (2023).

7. J. Yu, K. M. Rosso, S. M. Bruemmer, Charge and Ion Transport in NiO and Aspects of Ni Oxidation from First Principles. *J. Phys. Chem.* **116**, 1948-1954 (2012).

8. T. E. Karakasidis, G. A. Evangelakis, Cation Vacancy Diffusion on the NiO(001) Surface by Molecular Dynamics Simulations. *Surf. Sci.* **436**, 193-201 (1999).

9. M. L. Sushko, V. Alexandrov, D. K. Schreiber, K. M. Rosso, S. M. Bruemmer, Multiscale model of metal alloy oxidation at grain boundaries. *J Chem Phys* **142**, 214114 (2015).

10. D. Meng, B. Zheng, G. Lin, M. L. Sushko, Numerical Solution of 3D Poisson-Nernst-Planck Equations Coupled with Classical Density Functional Theory for Modeling Ion and Electron Transport in a Confined Environment. *Commun Comput Phys* **16**, 1298-1322 (2014).

11. D. K. Schreiber, M. J. Olszta, S. M. Bruemmer, Directly correlated transmission electron microscopy and atom probe tomography of grain boundary oxidation in a Ni-Al binary alloy exposed to high-temperature water. *Scripta Materialia* **69**, 509-512 (2013).

12. D. K. Schreiber, M. J. Olszta, S. M. Bruemmer, Grain boundary depletion and migration during selective oxidation of Cr in a Ni-5Cr binary alloy exposed to high-temperature hydrogenated water. *Scripta Materialia* **89**, 41-44 (2014).

13. V. Alexandrov, M. L. Sushko, D. K. Schreiber, S. M. Bruemmer, K. M. Rosso, Ab Initio Modeling of Bulk and Grain Diffusion in Ni Alloys. *Journal of Physical Chemistry Letters* **6**, 1618-1623 (2015).

14. B. J. Boyle, E. G. King, K. C. Conway, Heats of Formation of Nickel and Cobalt Oxides (Nio and Coo) of Combustion Calorimetry. *J Am Chem Soc* **76**, 3835-3837 (1954).

15. *CRC handbook of chemistry and physics:* . W. M. Haynes, Ed., 95th Edition (2014).

16. A. Navrotsky, L. Mazeina, J. Majzlan, Size-driven structural and thermodynamic complexity in iron oxides. *Science* **319**, 1635-1638 (2008).

17. S. J. Rothman, L. J. Nowicki, G. E. Murch, Self-Diffusion in Austenitic Fe-Cr-Ni Alloys. *J Phys F Met Phys* **10**, 383-398 (1980).
