# Supplementary material for: Selective atomic sieving across metal/oxide interface for super-oxidation resistance
Source: Nat Commun. 2024 Jul 21;15:6149. doi: 10.1038/s41467-024-50576-7 (PMC11271475; doi:10.1038/s41467-024-50576-7)
Supplement: Supplementary file 3 — Description of Additional Supplementary Files [file 41467_2024_50576_MOESM3_ESM.pdf]

Description of Additional Supplementary Files

Supplementary Movie 1 - *In situ* atomic-scale observation of oxidation process at the (111) and (001) surface of Ni-5Cr alloy beside one TB at 350 ℃ and pO_2 ~ 1×10^{-4} mbar. The movie was sped up 5 times.

Supplementary Movie 2 - *In situ* atomic-scale observation of oxide growth at the (111) surface of Ni-5Cr alloy at 350 ℃ and pO_2 ~ 1×10^{-4} mbar. The movie was sped up 10 times.

Supplementary Movie 3 - *In situ* atomic-scale observation of two layers oxide growth at the (001) surface of Ni-5Cr alloy at 350 ℃ and pO_2 ~ 1×10^{-4} mbar. The movie was sped up 20 times.
